# Evaluation of Algorithm Performance in ChIP-Seq Peak Detection

**DOI:** 10.1371/journal.pone.0011471

**Published:** 2010-07-08

**Authors:** Elizabeth G. Wilbanks, Marc T. Facciotti

**Affiliations:** 1 Graduate Group in Microbiology, University of California Davis, Davis, California, United States of America; 2 Department of Biomedical Engineering, University of California Davis, Davis, California, United States of America; 3 Genome Center, University of California Davis, Davis, California, United States of America; Radboud University Nijmegen, Netherlands

## Abstract

Next-generation DNA sequencing coupled with chromatin immunoprecipitation (ChIP-seq) is revolutionizing our ability to interrogate whole genome protein-DNA interactions. Identification of protein binding sites from ChIP-seq data has required novel computational tools, distinct from those used for the analysis of ChIP-Chip experiments. The growing popularity of ChIP-seq spurred the development of many different analytical programs (at last count, we noted 31 open source methods), each with some purported advantage. Given that the literature is dense and empirical benchmarking challenging, selecting an appropriate method for ChIP-seq analysis has become a daunting task. Herein we compare the performance of eleven different peak calling programs on common empirical, transcription factor datasets and measure their sensitivity, accuracy and usability. Our analysis provides an unbiased critical assessment of available technologies, and should assist researchers in choosing a suitable tool for handling ChIP-seq data.

## Introduction

*Ch*romatin *i*mmuno*p*recipitation followed by high-throughput *seq*uencing (ChIP-seq) is a technique that provides quantitative, genome-wide mapping of target protein binding events [1], [2]. Identifying putative protein binding sites from large, sequence-based datasets presents a bioinformatic challenge that has required considerable computational innovation despite the availability of numerous programs for ChIP-Chip analysis [3], [4], [5], [6], [7], [8], [9]. With the rising popularity of ChIP-seq, a demand for new analytical methods has led to the proliferation of available peak finding algorithms. Reviewing literature from the past three years, we noted 31 open source programs for finding peaks in ChIP-seq data (Table S1), in addition to the available commercial software. The sheer abundance of available software packages and technical variability with which they identify protein binding sites makes an assessment of the current methods timely. An appraisal of available analytical methods will better equip researchers to bridge the “next-generation gap” between sequencing and data analysis [10].

Recently, Pepke *et al*. published a review of the major steps in ChIP-seq analysis and detailed the algorithmic approaches of 12 available programs for detecting peaks (the signals of putative protein binding) from ChIP-seq data [11]. For clarity, we have provided a brief overview of the main algorithmic treatments of ChIP-seq data; however, our focus here is evaluative rather than purely descriptive. The purpose of this study is to provide an impartial analysis to help readers navigate the myriad of options. Laajala et al. [12] provide some metrics for evaluating different methods, but leave many areas unexplored. Our work offers several improved ways to assess algorithm performance and address the question: which of the available methods for ChIP-seq analysis should I consider using?

The ChIP protocol ideally produces a pool of DNA fragments that are significantly enriched for the target protein's binding site. High throughput sequencing of these fragments generates millions of short sequence ‘tags’ (generally 20 to 50 bp in length) that are subsequently mapped back to the reference genome. By recognizing regions in the genome with increased sequence coverage, ChIP-seq experiments identify the genomic coordinates of protein binding events. ChIP-seq peak finders must discriminate these true peaks in sequence coverage, which represent protein binding sites, from the background sequence.

**Figure 1:**
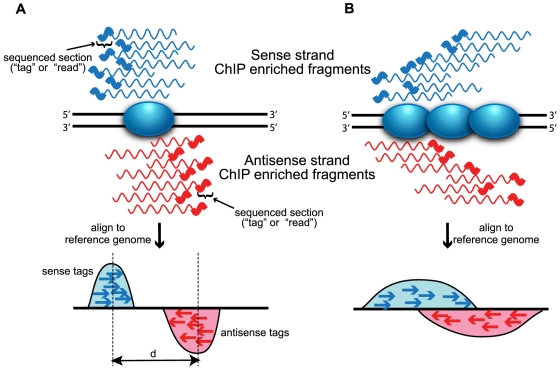
Strand-dependent bimodality in tag density. The 5′ to 3′ sequencing requirement and short read length produce stranded bias in tag distribution. The shaded blue oval represents the protein of interest bound to DNA (solid black lines). Wavy lines represent either sense (blue) or antisense (red) DNA fragments from ChIP enrichment. The thicker portion of the line indicates regions sequenced by short read sequencing technologies. Sequenced tags are aligned to a reference genome and projected onto a chromosomal coordinate (red and blue arrows). (**A**) Sequence-specific binding events (*e.g.* transcription factors) are characterized by “punctuate enrichment” [11] and defined strand-dependent bimodality, where the separation between peaks (d) corresponds to the average sequenced fragment length. Panel A was inspired by Jothi *et al*. [32]. (**B**) Distributed binding events (*e.g.* histones or RNA polymerase) produce a broader pattern of tag enrichment that results in a less defined bimodal pattern.

When examining tag density across the genome, it is important to consider that sequence tags can represent only the 5′-most end of the original fragment due to the inherent 5′ to 3′ nature of current generation of short-read sequencing instruments. This pattern results in a strand-dependent bimodality in tag density most evident in sequence-specific binding events, such as transcription factor-cis regulatory element binding (Figure 1). Most programs perform some adjustment of the sequence tags to better represent the original DNA fragment, either by shifting tags in the 3′ direction [13], [14], [15] or by extending tags to the estimated length of the original fragments [16], [17], [18], [19], [20], [21], [22], [23]. When the average fragment length can be accurately inferred (either computationally or empirically), the combined density will form a single peak where the summit corresponds closely to the binding site. If paired-end sequencing technologies are used, the fragment length can actually be measured directly allowing more precise determination of binding sites, a feature currently supported by only a handful of peak calling algorithms [13], [24], [25].

The first step in peak finding is to identify genomic regions with large numbers of mapped sequence tags. One approach to this task is to identify regions where extended sequence tags (XSETs) either overlap [19], [21], [26] or are found within some fixed clustering distance [16], [20], [22], [27]. Another commonly used method for finding enriched regions calculates the number of tags found in fixed width windows across the genome, an approach known as a sliding window algorithm [13], [15], [18], [23], [28], [29], [30], [31], [32]. As this histogram-type density estimator can produce edge effects dependent on the window or bin size, some programs instead employ a Gaussian kernel density estimator (G-KDE) that generates a continuous coverage estimate [14], [33], [34]. All these methods specify some minimum height criteria at which enrichment is considered significant, and some minimum spacing at which adjoining windows, clusters or local maxima (G-KDE) are merged into a single peak region.

Rather than searching for peaks in coverage, several methods leverage the bimodal pattern in the strand-specific tag densities to identify protein binding sites, either as their main scoring method [31], [32] or in an optional post-processing filtering step [19], [28]. Programs that use this signal exclusively, which we call “directional scoring methods,” are more appropriate for proteins that bind to specific sites (transcription factors), rather than more distributed binders, such as histones or RNA polymerase (Figure 1B).

CSDeconv, a recently published algorithm, uses both G-KDE and directional information in conjunction with a deconvolution approach, which enables detection of closely spaced binding sites [34]. Such an approach has been shown to have higher spatial resolution, though the intense computational demands limit the size of genomes that can be analyzed. Developed expressly for use on a bacterial genome, CSDeconv and programs like it may represent an excellent choice for microbial ChIP-seq experiments with only a few binding sites, small genome size and high sequence coverage.

More specialized programs for the analysis of RNA polymerase [35], [36] and epigenetic modifications [37], [38], [39], [40], [41] ChIP-seq also have been developed. These proteins bind DNA over larger regions, producing relatively broad, low-intensity peaks that can be difficult to detect. Though we focus on identifying transcription factor binding sites from ChIP-seq data, we mention these additional methods should readers find them appropriate for their specific experiments.

Peak finding programs must determine the number of tags (peak height) or directionality score that constitutes “significant” enrichment likely to represent a protein binding site. An *ad hoc* method for dealing with this issue is simply to allow users to select some threshold value to define a peak [16]. However, this simplistic approach does little to assist the user in assessing the significance of peaks and is prone to error. Other, more sophisticated methods assess the significance of sequence tag enrichment relative to the null hypothesis that tags are randomly distributed throughout the genome. The background modeled by the null hypothesis has been described previously using either a Poisson [15], [32] or negative binomial model [28], [30] parameterized based on the coverage of low-density regions in the ChIP sample. The actual background signal, however, shows decidedly non-random patterns [42], [43] and is only poorly modeled [44] by these methods, which have been demonstrated to systematically underestimate false discovery rates [31].

To account for the complex features in the background signal, many methods incorporate sequence data from a control dataset generated from fixed chromatin [16] or DNA immunoprecipitated with a nonspecific antibody [18], [42]. Control data can be used to make adjustments to the ChIP tag density prior to peak calling. Some methods implement background subtraction by calling peaks from the difference between ChIP and normalized control tag densities [15], [28], [31], while others use control data to identify and compensate large duplications or deletions in the genome [23].

Control tag densities are also used to assess the significance of peaks in the ChIP sample. One straightforward approach is to calculate the fold enrichment of ChIP tags over normalized control tags in candidate regions, to account for the fluctuating background signal [16], [18], [27], [32]. More statistical sophistication can be incorporated by employing statistical models parameterized from the normalized control sample to assess the significance of ChIP peaks. Different programs have implemented models of varying complexity, such as Poisson [14], [27], local Poisson [13], t-distribution [23], conditional binomial [15], [21], [28], and hidden Markov [29], [30] models. These statistical models are used primarily to assign each putative peak some significance metric, such as P-value, q-value, t-value or posterior probability. Control data can also be used to calculate empirical false discovery rates, by assessing the number of peaks in the control data (FDR  =  # control peaks / # ChIP peaks). Peaks are identified in control data either by swapping the ChIP and control data [13], [31], [34] or by partitioning the control data, if enough control sequence is available [14], [22]. The goal of all these different methods is to provide more rigorous filtering of false positives and accurate methods for ranking high confidence peak calls.

In this work, eleven peak calling algorithms are benchmarked against three empirical datasets from transcription factor ChIP-seq experiments. Our goal was to provide quantitative metrics for comparing available analysis programs based on the similarity of peaks called, sensitivity, specificity and positional accuracy. We find that many programs call similar peaks, though default parameters are tuned to different levels of stringency. While sensitivity and specificity of different programs are quite similar, more differences are noted in the positional accuracy of predicted binding sites.

## Results

### Overview

Peak calling programs employ a wide variety of algorithms to search for protein binding sites in ChIP-seq data; however, it remains unclear to what extent these differences in methodology and mathematical sophistication translate to substantial variation in performance. Definitively benchmarking the performance of different peak calling programs is challenging, since there exists no comprehensive list of all genomic locations bound by the target under the experimental conditions (true positives). In lieu of using empirical data, an *in silico* “spike-in” dataset can be generated by adding a known number of simulated ChIP peaks to control sequence [15]. However, such methods are, as yet, relatively unreliable due to challenges in mimicking the form and variability of empirical ChIP peaks.

We chose to test programs against three published transcription factor ChIP-seq datasets with controls: human neuron-restrictive silencer factor (NRSF) [16], growth-associated binding protein (GABP) [14], and hepatocyte nuclear factor 3α (FoxA1) [13]. Each of these transcription factors has a well-defined canonical binding motif (see Materials and Methods) that can be used to assess ChIP-seq peak quality and confidence. NRSF represents a particularly attractive test case, as the 21 bp canonical binding motif, NRSE2 [45], has been rigorously defined and is relatively high information content relative to the shorter GABP (12bp) and FoxA1 (10 bp) motifs. For further validation, we also make use of extensive lists of qPCR verified sites that are available for NRSF (83 sites) [45] and GABP (150 sites) [46] (available online as Dataset S1). While the empirical ChIP-seq datasets analyzed herein do not address interesting issues concerning biological replicates, we feel that interesting facet of ChIP-seq analysis has been studied expertly in previous publications [12], [21].

**Figure 2:**
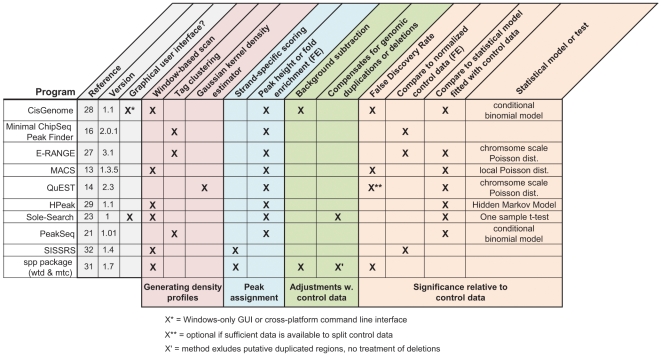
ChIP-seq peak calling programs selected for evaluation. Open-source programs capable of using control data were selected for testing based on the diversity of their algorithmic approaches and general usability. The common features present in different algorithms are summarized, and grouped by their role in the peak calling procedure (colored blocks). Programs are categorized by the features they use (Xs) to call peaks from ChIP-seq data. The version of the program evaluated in this analysis is shown for each program, as the feature lists can change with program updates.

Eleven peak calling methods capable of using control data were selected from the available open source programs, to represent the diversity of approaches in the different peak calling stages (Figure 2). To best approximate typical implementation by non-expert users, all programs were run with the default or recommended settings from the same desktop machine equipped with 4 Gb of RAM. While we note that some programs have many tunable parameters, we forgo extensive parameter optimization, which might have improved the results for some methods on the NRSF data, as this task is beyond the ken of most users.

#### Sensitivity

**Figure 3:**
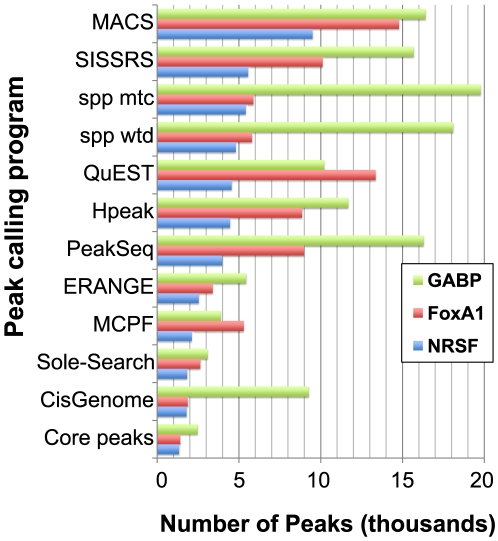
Quantity of peaks identified. Programs report different numbers of peaks, when run with their default or recommended settings on the same dataset. Number of reported peaks is shown for the GABP (green bars), FoxA1 (red bars) and NRSF (blue bars) datasets. To assess how different these peak lists were, those peaks identified by all 11 methods were calculated (core peaks).

**Figure 4:**
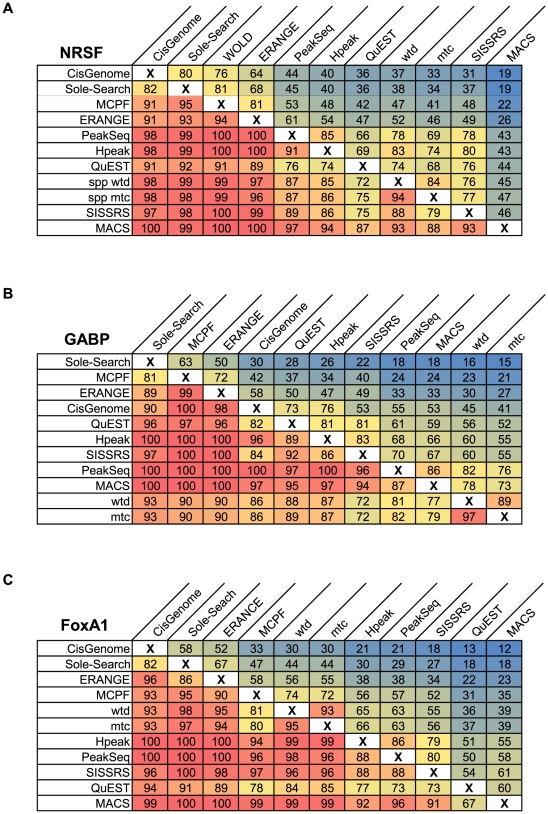
Pair-wise comparison of shared peaks. Pair-wise comparisons of the peak lists for **A**) NRSF, **B**) GABP and **C**) FoxA1 were conducted to determine the number of shared peaks between each pair of two methods. Each panel shows the percentage of total peaks from one method (column) that shared with another method (row). Programs in rows and columns are sorted by increasing number of peaks and entries are shaded by color gradients such that red represents the highest shared proportion and blue, the lowest.

For each of the three datasets, all peak callers reported a different number of peaks (Figure 3). The variation in the quantity of identified peaks indicates that default stringency levels are tuned differently among programs. A core set of peaks shared by all eleven programs was identified and found to comprise 75–80% of the smallest peak list for each ChIP-seq dataset (Figure 3). The set peaks shared by all methods suggests that smaller peak lists may, by and large, simply represent subsets of peaks called by programs with less stringent default parameters. Previous comparisons have offered only qualitative insights by examining the average overlap of a peak list will any different methods [12]. To more rigorously address this question, we conducted a series of pair-wise comparisons between the peak lists from each method to determine which peaks were shared. These comparisons are presented in Figure 4 as the percentage of each peak list (column) shared with another method (row). For all three datasets, a smaller peak list shared an average of 92% of its peaks with a larger peak list from a different method, whereas larger peak lists shared an average of only 45–55% of peaks with smaller peak lists. These figures indicate that more stringent peak lists from some programs are nearly completely contained within the larger number of calls by other methods, similar to the more general findings of Laajala *et al.* [12].

**Figure 5:**
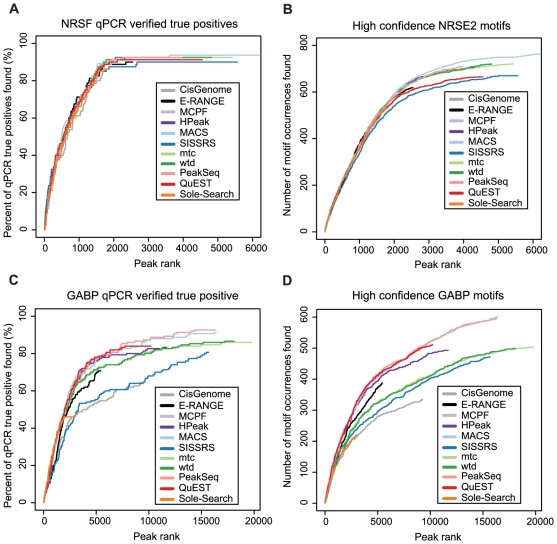
Sensitivity assessment. The percentage of qPCR verified positives that were detected by different programs is shown as a function of the increasing number of ranked peaks examined for the (**A**) NRSF dataset and its 83 qPCR-verified sites, or (**C**) the GABP dataset and its 150 qPCR-verified GABP binding sites. qPCR sites were classified as “found” if the center of the sites occurred within 250 bp of a program's predicted binding site (peak summit or peak region center). (**B**) Coverage of high confidence (FIMO p<1×10^−7^) NRSE2 motifs or (**D**) high confidence (FIMO p<1×10^−6^) GABP motifs throughout the human genome as a function of increasing ranked peaks examined. Motif occurrences were covered if the center of the motif occurred within 250 bp of a program's predicted binding site (peak summit or center of peak region).

This issue begs the question: what is gained by calling more peaks? To address this matter, we began by examining qPCR-validated true positive sites available for NRSF [45] and GABP [46]. The sensitivity of the methods was assessed by calculating the percentage of these true positives found by each program (Figure 5A,C). For NRSF, sensitivity of the different methods is remarkably similar up to the 1800 peak mark, after which SISSRS, E-RANGE and QuEST are slightly less sensitive. After 2500 peaks, the rate at which validated sites are discovered plateaus, yielding little gain in verified sites from the tail of the remaining peak lists. Sole-Search and CisGenome, which only identify about 1800 peaks, missed several positive sites picked up by programs calling more peaks. GABP showed more divergence in the sensitivity of the different programs to qPCR verified sites, with Sole-Search, CisGenome, and SISSRS falling well below the sensitivity of other algorithms. One of the most notable differences in performance between the NRSF and GABP datasets came from the Kharchenko's spp package, wtd and mtc, which were less sensitive in the GABP dataset. The decreased sensitivity of the spp methods on the GABP dataset may be caused by the broader enrichment regions noted in this dataset (see Figures S6, S7 and S8 and further discussion in the “Spatial Resolution” section). Directional scoring methods are known to be less useful for identifying broad enrichment signals, such as histone modification or RNA polymerase binding, due to blurring of the signal between the forward and reverse reads (Figure 1B).

Though high in confidence, the qPCR gold-standards cover only a handful of sites across the genome, perhaps limiting our ability to assess more subtle difference in sensitivity. To gain a more comprehensive picture of sensitivity between these methods, a whole genome scan for the presence of high confidence canonical binding motifs was conducted. This approach, which permits an assessment of sensitivity from a larger database, generated a list of more than 3000 potential NRSF and 6500 GABP binding sites. The coverage of these motif occurrences largely recapitulates the patterns seen with the qPCR binding site analysis, suggesting that the similarities observed with the high confidence qPCR database are not simply artifacts of the small sample size (Figure 5B,D). In summary, the sensitivity of all methods on the NRSF dataset remains remarkably similar over most of the peak-lists, while more noticeable differences emerge in examining the GABP data. The similarities from the NRSF data likely emerge from the fact that many algorithms may have been tested and trained on this same dataset, thereby optimizing their default settings. The differences seen with GABP highlight the potential variability in performance and seem to indicate that, for this dataset, directional scoring methods were less sensitive (SISSRS, mtc, wtd), corroborating the findings from our qPCR analysis.

It is important, however, to consider that high confidence motif sites represent *putative* binding sites for the transcription factor. Some sites may not be occupied under the experimental conditions and may not even be present in the cell line's genome, given that cell lines are prone to genomic instability. Thus, while the co-occurrence of motif instances and detected peaks likely represent true binding sites, the failure to identify a peak at a motif site has a several possible explanations.

#### Specificity

Assessing the rate of false positives in the peak lists is a challenging task. The available set of qPCR-determined negative sites for NRSF provides only 30 “true negatives”, defined as sites where enrichment was less than 3 fold [45]. By this standard, nine of eleven programs called a total of two putative false positives (CisGenome and QuEST found none). The same two “true negative” sites (chr20: 61280784–61280805 and chr6:108602345–108602365 in hg18) were identified by all nine programs. Although this could indicate some systematic bias in peak calling, Kharchenko *et al*. argue that, based on sequence tag distributions, these sites are likely bound by NRSF under the ChIP-seq experimental conditions (see Supplementary Fig. 9 from Kharchenko *et al*. [31]). Thus, we find these “negative” sites and their corollaries in the GABP dataset unreliable for assessing the specificity of the different programs using metrics such as a receiver operator curve (ROC), despite the fact that other groups have used this metric previously [12].

**Figure 6:**
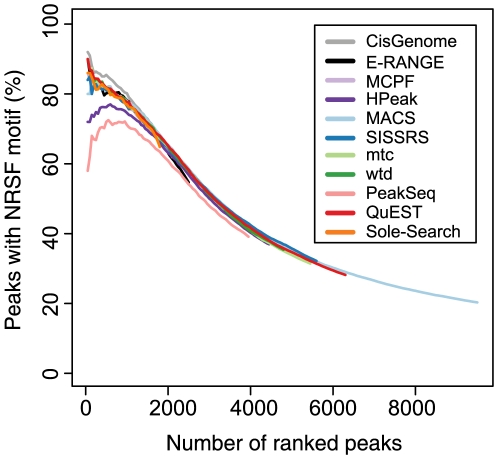
Ranking accuracy. Ranked peak lists were examined in increasing 50 peak intervals (50 peaks, 100 peaks, etc.). Peaks were deemed to contain a high confidence NRSE2 motif if a MAST search of the region surrounding the predicted binding site (peak summit or peak region center) yielded a motif within 500 bp (p<1×10^−6^) of the center. The percentage of peaks containing motifs was evaluated for each interval for all eleven methods.

In the absence of an appropriate dataset for rigorous false positive testing, many investigators prefer to examine a stringent set of binding sites. Thus, programs must provide accurate means for ranking peaks according to some confidence metric. To assess peak ranking accuracy, we calculated the rate of canonical motif occurrence for NRSF, GABP and FoxA1 within additive intervals of 50 peaks (top 50, top 100, top 150, etc; Figure 6 and Figures S1, S2). The percentage of peaks containing high confidence motifs decays with decreasing peak rank, suggesting that rank generally discriminates well between high confidence and lower confidence peaks. The performance of the different ChIP-seq methods at detecting high confidence NRSF binding sites is very similar; the percentage of motif-containing peaks varied by less than 3% with the exception of PeakSeq and HPeak. More variability is seen in the ranking of the top 50 peaks, though the methods still differ by only 10% when the outliers (PeakSeq and HPeak) are excluded. Over the first 2000 peaks, PeakSeq and HPeak detect between 10 and 20% fewer peaks with strong motifs than other algorithms. However, when a larger window (1 kb) surrounding the peak center is examined, the performance of these methods is comparable to other programs (Figure S3). This result suggests that both PeakSeq and HPeak identify peaks with lower positional resolution than other methods for the NRSF dataset. The decay of motif content in ranked peaks for the other two datasets were similarly tightly clustered, showing relatively little variation with the exception of slightly poorer performance for Sole-Search in the GABP dataset and QuEST in the FoxA1 dataset (Figure S1 and S2, respectively). While changes in the significance threshold set for defining a motif occurrence impacted absolute percentage of peaks containing motifs, such changes did not alter the performance of the programs relative to one another (Figure S5). Another interesting point with regards to peak ranking is that the different statistics provided by the same program can produce substantially different rankings, with variable success at determining high-quality peaks (Figure S4).

This peak ranking analysis provides considerably more practical information to the user than does the motif analysis conducted by Laajala et al. [12], which simply reports the average significance of motif overlap with all peaks. Our results support their general conclusion that the whole peak lists from all programs show significant proportion of the canonical binding motif and also demonstrate the significance of peak rank in recovering high confidence motif sites.

We note that the absence of a strong motif occurrence does not definitively classify peaks as false positives, as some such peaks could represent true binding sites with weak or non-canonical binding motifs. Nonetheless, high confidence motif occurrences within peaks are a good indicator of an actual binding event and can be used to assess how well peak ranking identifies the most confident binding sites. Furthermore, previous studies of non-canonical motifs suggest that these sites makes up a relatively minor fraction of overall motif occurrences [16].

Given the vagaries of ChIP enrichments, it is important to consider the robustness specificity in peak calling with “noisy” data. Less efficient ChIP enrichments will produce datasets with a larger ratio of non-specific background sequence to ChIP-targeted sequence. Such datasets will thus be characterized by higher background noise, lower peaks and under-sampling of low-intensity peaks. The complexity of features in the background sequence (discussed in Introduction) makes modeling “noise” features extremely challenging. We have simulated noisy datasets *in silico* by removing randomly sampled ChIP reads from Johnson *et al.* 's NRSF dataset and introducing an equal number of reads from the background data. Datasets were simulated where the noisy ChIP sample was composed 10%, 30% and 50% reads sampled from the background control dataset. These increasingly noisy datasets are meant to simulate decreasing efficiency ChIP enrichments with the same sequencing coverage.

As expected, the number of peaks called decreases in simulations of less efficient ChIP (Figure S6). The size of the decrease tended to be most marked for programs that called larger peak lists, suggesting that it was the smaller peaks were lost in the noise. This conclusion was borne out in by searching for canonical motifs in the ranked peak lists from our simulated noisy data. Few differences were observed between variable noise datasets in the motif content of ranked peaks (Figure S7), indicating that though all programs lost some peaks in the noise, they tended not to increase spurious peak calls. QuEST showed the most notable decay of motif content in noisier datasets, likely because this algorithm's background filtering method relies on larger control datasets. In noisier simulations, HPeak and PeakSeq showed increasing motif content in the top 500 peaks, such that it seems that their ranking algorithms performed better on noisier datasets. Further investigation is needed to discover the origin of this phenomenon, though we suspect that this may be due to better spatial precision in their identifications. In summary, however, we find few substantial differences between the performance of these programs on our simulated datasets at increasing noise thresholds.

#### Spatial resolution

In addition to discriminating the true binding sites, a ChIP-seq peak finder should identify that binding site with some degree of precision to facilitate the location of DNA-protein binding. The width of identified peaks can be an important consideration for *de novo* motif searches of peaks identified by ChIP-seq, since extraneous sequence around the true protein binding adds significant noise that can obscure the motif signal. Most programs will report a peak region of variable width, given by start and stop coordinates. However, directionality-scoring methods tend to report either narrow fixed width peaks (SISSRS) or single coordinate peaks (spp package), rather than the wider regions reported by other methods. For both the FoxA1 and NRSF datasets, the median peak width was between 250 and 400 bp for all methods reporting peak width ranges, with the exception of CisGenome which had smaller median peak width (72 bp for NRSF and 90 bp for GABP; Figure S8 and S9). In contrast, peaks called from the GABP dataset tended to be wider, with median peak widths ranging from 300 to 800 bp, excepting CisGenome which was only 90 bp (Figure S10). This observed variance between datasets emerges either from actual differences in transcription factor binding (GABP binding in a more distributed manner), from variation in the preparation of samples (such as differences in antibody specificity or size selection during the preparation of the sequencing library) or a combination of such factors.

**Figure 7:**
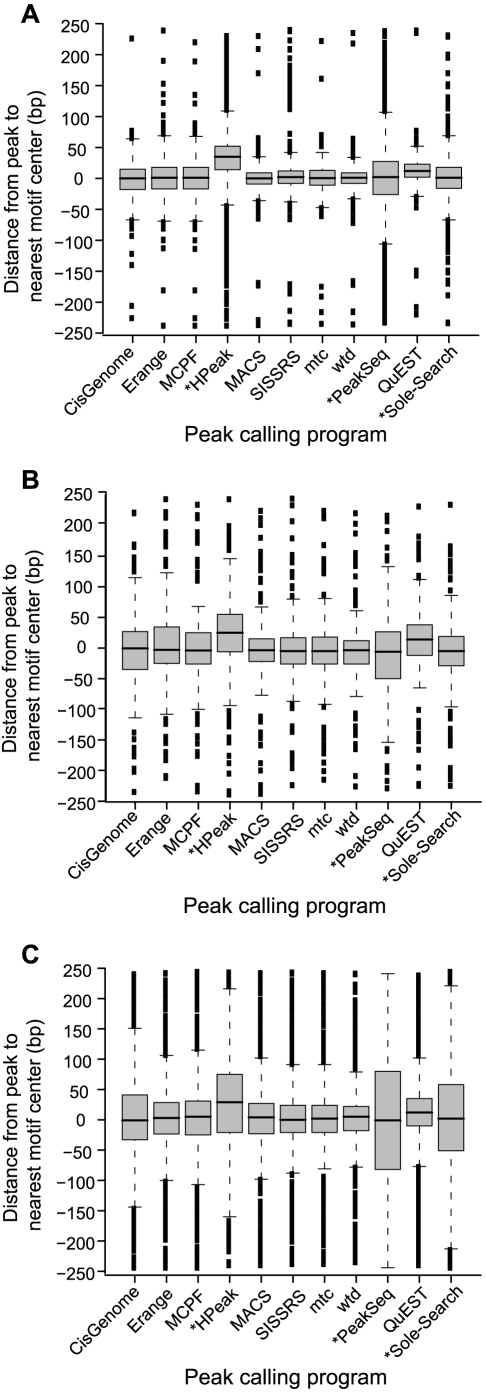
Positional accuracy and precision. The distance between the predicted binding site and high confidence motif occurrences within 250 bp was calculated for different peak calling programs in the (**A**) NRSF, (**B**) FoxA1, and (**C**) GABP datasets. Negative distances indicate that the motif was found before the peak coordinate (*e.g.* a motif centered at chr1∶1000 and predicted binding site at chr:1050 corresponds to a distance of −50bp). The variation in distances from predicted binding sites to motif center is presented as a box-and-whisker plot for each program. Starred programs (*) indicate that these methods did not provide a predicted binding coordinate; so binding positions were estimated as the center of the reported peak region. Exact numbers are available in Table S3.

In general, programs also provide an estimate of the exact binding position, given as a single coordinate calculated either as the highest point of tag coverage in the peak or by some other scoring metric. This coordinate is meant to aid the researcher in honing in on section of DNA originally cross-linked by the target protein during the ChIP-enrichment step. Though there is no single nucleotide at which cross-linking occurs, this estimate is meant to facilitate the precise discovery of *cis*-regulatory elements [11]. To assess the positional accuracy of these estimates made by different programs, the distance was calculated between each predicted binding coordinate and the centers of high confidence binding motifs within 250 bp (Figure 7, Table S3). Binding positions were estimated as the center of the reported peak region, if the program did not provide a predicted binding coordinate (HPeak, PeakSeq and Sole-Search; starred in Figure 7). Unsurprisingly, all three datasets revealed that these centered estimates provided much less positional resolution than the precise predictions of binding positions by other programs.

For all programs, the positional accuracy was lower for the GABP dataset (Figure 7C) than for either FoxA1 or NRSF. Keeping in mind the wider peak regions called for the GABP dataset, we conclude that the signal from binding events in this GABP dataset is likely broader, which makes precise estimation of the binding location more challenging. However, the same trends in each program's positional accuracy were observed throughout the three datasets, despite changes in the absolute magnitude. The predictions for QuEST and HPeak were both consistently shifted downstream from the nearest high confidence motif occurrence (3′ direction, positive shift in Figure 7), indicating some unknown, systematic bias in these unrelated algorithms. MACS and the three directionality dependent methods (SISSRS and Kharchenko's wtd and mtc programs) were provided some of the best spatial resolution of binding events. The success of directionality scoring methods follows logically from their search strategy which, unlike other methods, hinges upon identifying a single “transition point” between the tag densities on the sense and antisense strands.

## Discussion

Selecting a peak detection algorithm is central to ChIP-seq experimental studies. Though the algorithmic details may seem arcane to many biologists, computational analysis is the key to leveraging meaningful information about biology from sequence-based data. We demonstrate that eleven ChIP-seq analysis programs of varying algorithmic complexity identify protein binding sites from common empirical datasets with remarkably similar performance with regards to sensitivity and specificity. We find few substantial differences between the performance of these programs on our simulated datasets at increasing noise thresholds. A more complete analysis of the origin of noise and improved metrics for determining the noisiness of datasets would certainly benefit future in ChIP-seq experiments.

The programs differed most significantly in the spatial resolution of their estimates for the precise binding region. The best estimates of precise binding location were provided by Kharchenko *et al.* 's ChIP-seq processing pipeline (spp) [31], which uses directionality scoring, followed shortly by Zhang *et al*. 's popular MACS program [13]. These tools would be an excellent choice especially for applications such as *de novo* motif discovery in regions with multiple motifs, where it is important to accurately minimize sequence search space. We base our observations on the analysis of sequence data generated exclusively from transcription factor ChIPs. Since different physical factors inherently influence peak profiles from non-transcription factor ChIPs (e.g. RNA polymerase, epigenetic modifications) we expect algorithm performance to differ significantly for such datasets. Several algorithms have been written to specifically address this issue and should be chosen in lieu of those evaluated herein if non-transcription factors are being studied [35], [36], [37], [38], [39], [40], [41].

Given the similarities in performance, the implementation and general usability of the different programs is an important factor in choosing an analysis tool (Figure 2). Most programs are run from the command line and require variable degrees of data formatting and computation expertise to implement. Kharchenko *et al.* 's ChIP-seq processing pipeline (spp) is run as a package from within the statistical program R, which facilitates data visualization and downstream analysis for the statistically-inclined user. CisGenome [28] and Sole-Search [23] can be implemented with a graphical user interface (GUI) which is important consideration for the bench-top biologist. CisGenome provides an integrated platform for ChIP-chip and ChIP-seq analysis, combined with downstream motif finding and an integrated genome browser; however, the CisGenome GUI is currently restricted to the Windows platform. Sole-Search runs a cross-platform compatible Java-based GUI that locally formats and compresses the data before uploading it to a web-server for remote analysis, a useful feature for users with limited computing resources and expertise.

An important consideration for ChIP-seq peak detection concerns the desired balance between sensitivity and specificity in compiling the final candidate peak list. Depending on the biological question, the user may want to examine either a stringent list of the most-confident peaks or a more comprehensive set of peaks that may include more false positives. It is crucial that this balance of stringency and sensitivity be a tunable to the needs of the user. Changing various parameters in each program and re-running the analysis can adjust the number of peaks reported. Alternatively, the user can simply rank called peaks according to some peak statistic (such as number or tags, fold enrichment over background, or p-value) and analyze only the top *n*-peaks where *n* is adjusted according to the researchers' desired stringency. Relative to previous reviews of ChIP-seq algorithms [12], our analysis provides considerably more resolution throughout the peak lists (50 peak intervals) and offers a better glimpse at how peak “quality” declines with decreasing rank.

We have demonstrated that ChIP-seq peak callers need not be overly sophisticated in their algorithmic approach to achieve comparable performance identifying relatively stringent lists of binding sites. While our assessment suggests that improvements in peak calling specificity and sensitivity are possible, it seems clear that the field faces a conundrum. It is challenging to rigorously assess subtle improvements due to the scarcity and unreliability of verified binding sites for any ChIP-seq dataset. Furthermore, without adequate verification data for false positive testing, the decision of how many peaks to evaluate as putative binding sites remains a matter of biological intuition combined with trial and error, despite layers of statistical sophistication. Recent studies [21], [22] suggest that using full biological replicates in ChIP-seq experiments may provide the most reliable manner of filtering false positives from true binding sites, a practice already encouraged by several groups such as the ENCODE consortium [21], [47]. We suggest that rather than focus solely on algorithmic development, equal or better gains could be made through careful consideration of experimental design and further development of sample preparations to reduce noise in the datasets.

## Methods

### Chip-seq data

Raw sequencing reads for the NRSF dataset [16] (kindly provided by A. Mortazavi) and GABP dataset [14] (downloaded from the QuEST website, http://mendel.stanford.edu/SidowLab/downloads/quest/) were aligned to the human genome (NCBI Build 36.1) using Bowtie [48]. The FoxA1 dataset [13] was downloaded as reads aligned to the human genome (NCBI Build 36.1) from the MACS website (http://liulab.dfci.harvard.edu/MACS/Sample.html). The datasets had the following number of uniquely mapped sequence reads, NRSF ChIP: 2,088,238 with 3,079,013 input control reads; GABP ChIP: 7,829,282 with 17,299,213 input control reads; FoxA1 ChIP: 3,909,805 with 5,233,683 input control reads.

### Program implementation

Unless otherwise specified all peak calling programs were run with default or recommended setting from a 2.66 GHz Intel Core i5 MacOSX desktop machine equipped with 4 GB of RAM. CisGenome GUI mode was tested on a virtualized instance of the Windows OS running from the aforementioned Mac. The Sole-Search program runs by default via submission to a web-server. Peaks with overlapping coordinates from different program's peak lists were determined by pair-wise comparison using BEDTools [49].

#### Ranking peaks

Peak lists that were not ranked automatically by programs were sorted according to peak characteristics reported by each program (Supplemental Table S2). PeakSeq and CisGenome return ranked lists by default. The Minimal ChIP-seq Peak Finder peak list was sorted by the number of reads in the cluster, E-RANGE by the fold enrichment and then by p-value, HPeak by peak's maximum coverage, SISSRS by fold enrichment and then p-value, MACS by the 10*−log_10_(p-value) and then by fold enrichment, the wtd and mtc methods from the spp package by the false discovery rate and then by the score, and Sole-Search by the peak's read count and then by the effect size. The regions in the QuEST peak list were sorted by q-value rank and only the most significant peak in each region was retained as QuEST's estimate of the exact binding site.

#### Positional Accuracy and Peak Motif Content

All motif searching was conducted using programs from the MEME/MAST package [50] and the following instances of the TF's canonical binding motif: the well-defined NRSE2 motif [45] was used for NRSF, while the TRANSFAC [51] database motifs were used for GABP (M00341) and FoxA1 (M01261). An exact binding site prediction was available from all programs except PeakSeq, Sole-Search and HPeak (though HPeak 2.1 has this feature, this version was available only for the Linux OS at the time of writing). In the absence of a predicted binding site, the center of each peak region was substituted as an exact binding site prediction. Regions 250 base pairs upstream and downstream from the predicted binding site were searched using MAST [50] for the high confidence hits of the canonical motif for the TF. Positional accuracy was assessed for the top 1500 peaks from each method as the distance from the predicted binding site to the center of the closest high confidence motif occurrence within 250 bp. The percentage of peaks containing at least one significant motif within 250 bp of the predicted binding site was calculated for additive 50 peak increments throughout the each program's ranked list of peaks.

#### Sensitivity analysis

Eighty-three qPCR validated NRSF-positive sites were obtained from Mortazavi *et al*. [45] and 150 qPCR GABP-positive sites were found in Collins *et al.* [46]. A set of 3002 high confidence NRSE2 motif [45] occurrences in the human genome were identified by FIMO [50] search of human genome build NCBI Build 36.1, using cutoff of p <1×10^−7^. For GABP, a set of 6670 motif occurrences in the human genome were identified by FIMO [50] search using a cutoff of p <1×10^−6^. The corresponding FIMO search for the FoxA1 motif returned >40,000 highly repetitive motif occurrences, having only 2 distinct p-values. Unable to define a subset of high confidence motifs in the whole genome, sensitivity analysis was not conducted for FoxA1. For NRSF and GABP, the number of high confidence motif occurrences found within peak regions was determined for 1-peak increments throughout each ranked peak list, using a combination of custom Perl scripts and BEDTools [49].

## Supporting Information

Table S1 Survey of open-source ChIP-Seq analysis programs. References that also appear in the main text are numbered accordingly. Supplementary references are indicated by S1 (etc) and NA indicates that the program has not yet been published. Websites hosting the code are provided for each method, unless the code was not publicly released at time of writing (usually available on request from authors).
(0.07 MB DOC)

Table S2 Methods used to rank peak lists from different programs. If programs returned a sorted peak list by default, no further sorting was conducted (NA). Secondary sorting method was used to break ties following the primary sorting.
(0.03 MB DOC)

Table S3 Median and standard deviation of positional accuracy data. Median and standard deviation of the distance from estimated binding sites to the nearest high confidence motif occurrence, measured in base pairs. Measurements conducted for the top 1500 peaks in each peak list. Represented graphically in Figure 7 of the main text.
(0.04 MB DOC)

Dataset S1 qPCR verified sites for NRSF and GABP. qPCR sites studied from previous publications are presented as regions in hg18 coordinates. Data available in separate tabs of this multitab Excel file.
(0.03 MB XLS)

Figure S1 GABP ranking accuracy. Ranked peak lists were examined in increasing 50 peak intervals (50 peaks, 100 peaks, etc.). Peaks were deemed to contain a high confidence GABP motif if a MAST search of the region surrounding the predicted binding site (peak summit or peak region center) yielded a motif within 500 bp (p<1×10^−4^) of the center. The percentage of peaks containing motifs was evaluated for each interval for all eleven methods.
(0.56 MB EPS)

Figure S2 FoxA1 ranking accuracy. Ranked peak lists were examined in increasing 50 peak intervals (50 peaks, 100 peaks, etc.). Peaks were deemed to contain a high confidence FoxA1 motif if a MAST search of the region surrounding the predicted binding site (peak summit or peak region center) yielded a motif within 500 bp (p<1×10^−4^) of the center. The percentage of peaks containing motifs was evaluated for each interval for all eleven methods.
(0.54 MB EPS)

Figure S3 NRSF Ranking accuracy revisited (1 kb regions). Ranked peak lists were examined in increasing 50 peak intervals (50 peaks, 100 peaks, etc.). Peaks were deemed to contain a high confidence NRSE2 motif if a MAST search of the region surrounding the predicted binding site (peak summit or peak region center) yielded a motif within 1 kb bp (p<1×10^−6^) of the center. The percentage of peaks containing motifs was evaluated for each interval for all eleven methods for the top 2000 peaks.
(0.51 MB EPS)

Figure S4 Different confidence metrics yield different rankings. Peak confidence measures provided by the same program can produce quite different rankings with different proportions of high confidence motifs. Ranking of MACS peak list by three different confidence measures (1st in figure legend indicates the primary means of sorting, the 2nd measure is used to break any ties). Analysis as in Figures S1–S3.
(0.82 MB EPS)

Figure S5 Motif stringency thresholds. Using either A) less stringent (p<1×10^−5^) or B) more stringent (p<1×10^−8^) thresholds for defining “significant” NRSE2 motifs found by MAST search within 500 bp of the peak did not change the relative ranking of the eleven tested methods. Compare with main text Figure 6.
(0.50 MB EPS)

Figure S6 Peaks called from simulated. A) Number of peaks called in from normal and simulated datasets at different noise levels. B) Percent decrease in the number of peaks called by each program was calculated as the difference between the normal and simulated datasets divided by the size of normal dataset.
(1.08 MB EPS)

Figure S7 Motif content in ranked peaks from simulated noisy datasets. Panels show the change in motif content throughout the peak lists in Johnson et al. 's unpertubed ChIP sample and 10–50% noise introduction from background sequence for each program.
(0.08 MB PDF)

Figure S8 Variation in width of peak regions reported by different ChIP-Seq peak callers for the NRSF dataset. The width of each peak was calculated as the difference between start and stop coordinates. Continuous density plots were generated to display the frequency with which different peak widths were observed in the lists reported by different peak calling programs. SISSRS and spp package programs (mtc and wtd) were not included as these methods report fixed width or single coordinate, respectively.
(2.38 MB EPS)

Figure S9 Variation in width of peak regions reported by different ChIP-Seq peak callers for the FoxA1 dataset. The width of each peak was calculated as the difference between start and stop coordinates. Continuous density plots were generated to display the frequency with which different peak widths were observed in the lists reported by different peak calling programs. SISSRS and spp package programs (mtc and wtd) were not included as these methods report fixed width or single coordinate, respectively.
(3.40 MB EPS)

Figure S10 Variation in width of peak regions reported by different ChIP-Seq peak callers for the GABP dataset. The width of each peak was calculated as the difference between start and stop coordinates. Continuous density plots were generated to display the frequency with which different peak widths were observed in the lists reported by different peak calling programs. SISSRS and spp package programs (mtc and wtd) were not included as these methods report fixed width or single coordinate, respectively.
(3.90 MB EPS)

## References

[1] Park PJ (2009). ChIP-seq: advantages and challenges of a maturing technology.. Nat Rev Genet.

[2] Barski A, Zhao K (2009). Genomic location analysis by ChIP-Seq.. J Cell Biochem.

[3] Reiss DJ, Facciotti MT, Baliga NS (2008). Model-based deconvolution of genome-wide DNA binding.. Bioinformatics.

[4] Qi Y, Rolfe A, MacIsaac KD, Gerber GK, Pokholok D (2006). High-resolution computational models of genome binding events.. Nat Biotechnol.

[5] Johnson WE, Li W, Meyer CA, Gottardo R, Carroll JS (2006). Model-based analysis of tiling-arrays for ChIP-chip.. Proc Natl Acad Sci USA.

[6] Boyer LA, Lee TI, Cole MF, Johnstone SE, Levine SS (2005). Core transcriptional regulatory circuitry in human embryonic stem cells.. Cell.

[7] Buck MJ, Nobel AB, Lieb JD (2005). ChIPOTle: a user-friendly tool for the analysis of ChIP-chip data.. Genome Biol.

[8] Ji H, Wong WH (2005). TileMap: create chromosomal map of tiling array hybridizations.. Bioinformatics.

[9] Kim TH, Barrera LO, Zheng M, Qu C, Singer MA (2005). A high-resolution map of active promoters in the human genome.. Nature.

[10] McPherson JD (2009). Next-generation gap.. Nat Methods.

[11] Pepke S, Wold B, Mortazavi A (2009). Computation for ChIP-seq and RNA-seq studies.. Nat Methods.

[12] Laajala TD, Raghav S, Tuomela S, Lahesmaa R, Aittokallio T (2009). A practical comparison of methods for detecting transcription factor binding sites in ChIP-seq experiments.. BMC Genomics.

[13] Zhang Y, Liu T, Meyer C, Eeckhoute J, Johnson D (2008). Model-based Analysis of ChIP-Seq (MACS).. Genome Biology.

[14] Valouev A, Johnson DS, Sundquist A, Medina C, Anton E (2008). Genome-wide analysis of transcription factor binding sites based on ChIP-Seq data.. Nat Methods.

[15] Nix D, Courdy S, Boucher K (2008). Empirical methods for controlling false positives and estimating confidence in ChIP-Seq peaks.. BMC Bioinformatics.

[16] Johnson D, Mortazavi A, Myers R, Wold B (2007). Genome-Wide Mapping of in Vivo Protein-DNA Interactions.. Science.

[17] Robertson G, Hirst M, Bainbridge M, Bilenky M, Zhao Y (2007). Genome-wide profiles of STAT1 DNA association using chromatin immunoprecipitation and massively parallel sequencing.. Nat Methods.

[18] Chen X, Xu H, Yuan P, Fang F, Huss M (2008). Integration of external signaling pathways with the core transcriptional network in embryonic stem cells.. Cell.

[19] Fejes A, Robertson G, Bilenky M, Varhol R, Bainbridge M (2008). FindPeaks 3.1: a tool for identifying areas of enrichment from massively parallel short-read sequencing technology.. Bioinformatics.

[20] Kallin E, Cao R, Jothi R, Xia K, Cui K (2009). Genome-Wide uH2A Localization Analysis Highlights Bmi1-Dependent Deposition of the Mark at Repressed Genes.. PLoS Genet.

[21] Rozowsky J, Euskirchen G, Auerbach RK, Zhang ZD, Gibson T (2009). PeakSeq enables systematic scoring of ChIP-seq experiments relative to controls.. Nat Biotechnol.

[22] Tuteja G, White P, Schug J, Kaestner KH (2009). Extracting transcription factor targets from ChIP-Seq data.. Nucleic Acids Res.

[23] Blahnik KR, Dou L, O'Geen H, McPhillips T, Xu X (2009). Sole-Search: an integrated analysis program for peak detection and functional annotation using ChIP-seq data.. Nucleic Acids Res.

[24] Wang C, Xu J, Zhang D, Wilson ZA (2010). An effective approach for identification of in vivo protein-DNA binding sites from paired-end ChIP-Seq data.. BMC Bioinformatics.

[25] Wilder S (2010). http://www.ebi.ac.uk/~swilder/SWEMBL/.

[26] Barski A, Cuddapah S, Cui K, Roh TY, Schones DE (2007). High-resolution profiling of histone methylations in the human genome.. Cell.

[27] Mortazavi A, Williams BA, McCue K, Schaeffer L, Wold B (2008). Mapping and quantifying mammalian transcriptomes by RNA-Seq.. Nat Methods.

[28] Ji H, Jiang H, Ma W, Johnson D, Myers R (2008). An integrated software system for analyzing ChIP-chip and ChIP-seq data.. Nat Biotechnol.

[29] Qin S, Shen J (2009). HPeak: A HMM-based algorithm for defining read-enriched regions from massive parallel sequencing data.. http://www.sph.umich.edu/csg/qin/HPeak.

[30] Spyrou C, Stark R, Lynch AG, Tavare S (2009). BayesPeak: Bayesian analysis of ChIP-seq data.. BMC Bioinformatics.

[31] Kharchenko PV, Tolstorukov MY, Park PJ (2008). Design and analysis of ChIP-seq experiments for DNA-binding proteins.. Nat Biotechnol.

[32] Jothi R, Cuddapah S, Barski A, Cui K, Zhao K (2008). Genome-wide identification of in vivo protein-DNA binding sites from ChIP-Seq data.. Nucleic Acids Res.

[33] Boyle AP, Guinney J, Crawford GE, Furey TS (2008). F-Seq: A Feature Density Estimator for High-Throughput Sequence Tags.. Bioinformatics.

[34] Lun DS, Sherrid A, Weiner B, Sherman DR, Galagan JE (2009). A blind deconvolution approach to high-resolution mapping of transcription factor binding sites from ChIP-seq data.. Genome Biol.

[35] Taslim C, Wu J, Yan P, Singer G, Parvin J (2009). Comparative Study on ChIP-seq Data: Normalization and Binding Pattern Characterization.. Bioinformatics.

[36] Feng W, Liu Y, Wu J, Nephew K, Huang T (2008). A Poisson mixture model to identify changes in RNA polymerase II binding quantity using high-throughput sequencing technology.. BMC Genomics.

[37] Xu H, Wei C-L, Lin F, Sung W-K (2008). An HMM approach to genome-wide identification of differential histone modification sites from ChIP-seq data.. Bioinformatics.

[38] Hon G, Ren B, Wang W (2008). ChromaSig: A Probabilistic Approach to Finding Common Chromatin Signatures in the Human Genome.. PLoS Comput Biol.

[39] Zang C, Schones DE, Zeng C, Cui K, Zhao K (2009). A clustering approach for identification of enriched domains from histone modification ChIP-Seq data.. Bioinformatics.

[40] Johannes F, Wardenaar R, Colome-Tatche M, Mousson F, de Graaf P (2010). Comparing genome-wide chromatin profiles using ChIP-chip or ChIP-seq.. Bioinformatics.

[41] Xu H, Handoko L, Wei X, Ye C, Sheng J (2010). A Signal-Noise Model for Significance Analysis of ChIP-seq with Negative Control.. Bioinformatics.

[42] Auerbach RK, Euskirchen G, Rozowsky J, Lamarre-Vincent N, Moqtaderi Z (2009). Mapping accessible chromatin regions using Sono-Seq.. Proc Natl Acad Sci USA.

[43] Vega VB, Cheung E, Palanisamy N, Sung W-K (2009). Inherent signals in sequencing-based chromatin-immunoprecipitation control libraries.. PLoS ONE.

[44] Zhang Z, Rozowsky J, Snyder M, Chang J, Gerstein M (2008). Modeling ChIP sequencing *in silico* with applications.. PLoS Comput Biol.

[45] Mortazavi A, Leeper Thompson EC, Garcia ST, Myers RM, Wold B (2006). Comparative genomics modeling of the NRSF/REST repressor network: from single conserved sites to genome-wide repertoire.. Genome Res.

[46] Collins PJ, Kobayashi Y, Nguyen L, Trinklein ND, Myers RM (2007). The ets-related transcription factor GABP directs bidirectional transcription.. PLoS Genet.

[47] Rosenbloom KR, Dreszer TR, Pheasant M, Barber GP, Meyer LR (2009). ENCODE whole-genome data in the UCSC Genome Browser.. Nucleic Acids Res.

[48] Langmead B, Trapnell C, Pop M, Salzberg SL (2009). Ultrafast and memory-efficient alignment of short DNA sequences to the human genome.. Genome Biol.

[49] Quinlan AR, Hall IM (2010). BEDTools: a flexible suite of utilities for comparing genomic features.. Bioinformatics.

[50] Bailey TL, Boden M, Buske FA, Frith M, Grant CE (2009). MEME SUITE: tools for motif discovery and searching.. Nucleic Acids Res.

[51] Wingender E, Dietze P, Karas H, Knuppel R (1996). TRANSFAC: a database on transcription factors and their DNA binding sites.. Nucleic Acids Res.

