# Supplementary material for: Evaluation of Algorithm Performance in ChIP-Seq Peak Detection
Source: PLoS One. 2010 Jul 8;5(7):e11471. doi: 10.1371/journal.pone.0011471 (PMC2900203; doi:10.1371/journal.pone.0011471)
Supplement: Table S1 — Survey of open-source ChIP-Seq analysis programs. References that also appear in the main text are numbered accordingly. Supplementary references are indicated by S1 (etc) and NA indicates that the program has not yet been published. Websites hosting the code are provided for each method, unless the code was not publicly released at time of writing (usually available on request from authors). (0.07 MB DOC) [file pone.0011471.s001.doc]

**Supplementary Table 1.** Survey of open-source ChIP-Seq analysis programs. References that also appear in the main text are numbered accordingly. Supplementary references are indicated by S1 (etc) and NA indicates that the program has not yet been published. Websites hosting the code are provided for each method, unless the code was not publicly released at time of writing (usually available on request from authors).

| **Program** | **Ref.** | **Website** | **Language** |
| --- | --- | --- | --- |
| MACS | 13 | <http://liulab.dfci.harvard.edu/MACS/> | Python |
| QuEST | 14 | <http://mendel.stanford.edu/SidowLab/downloads/quest/> | Perl |
| Useq | 15 | <http://useq.sourceforge.net/> | Perl, C |
| Minimal ChIPSeq PeakFinder | 16 | <http://woldlab.caltech.edu/html/software> | Python |
| XSET | 17 | Not publicly released |  |
| "The ChipSeq  Peak Finder" | 18 | <http://cmb.gis.a-star.edu.sg/ChIPSeq/paperChIPSeq.htm> | C |
| FindPeaks | 19 | <http://vancouvershortr.sourceforge.net/> | java |
| TIROE | 20 | Not publicly released |  |
| PeakSeq | 21 | <http://www.gersteinlab.org/proj/PeakSeq/> | Perl / C |
| GLITR | 22 | <http://web.me.com/kaestnerlab1/GLITR/> | Perl, Python |
| Sole-Search | 23 | <http://chipseq.genomecenter.ucdavis.edu/cgi-bin/chipseq.cgi> | Perl, java |
| E-RANGE | 27 | <http://woldlab.caltech.edu/rnaseq/> | Python |
| CisGenome | 28 | <http://www.biostat.jhsph.edu/~hji/cisgenome/> | C/C++ |
| BayesPeak | 30 | <http://www.compbio.group.cam.ac.uk/Resources/BayesPeak/csbayespeak.html> | Perl / C |
| spp (R package) | 31 | <http://compbio.med.harvard.edu/Supplements/ChIP-seq/> | R |
| SISSRS | 32 | <http://sissrs.rajajothi.com/> | Perl |
| F-Seq | 33 | <http://www.genome.duke.edu/labs/furey/software/fseq> | java |
| SIPeS | 24 | http://gmdd.shgmo.org/Computational-Biology/ChIP-Seq/download/SIPeS | C |
| CSDeconv | 34 | http://www.unisa.edu.au/maths/phenomics/csdeconv/ | MATLAB R2009a |
| SWEMBL | 25 | <http://www.ebi.ac.uk/~swilder/SWEMBL/> | C |
| GeneTrack | S1 | <http://code.google.com/p/genetrack/> |  |
| HPeak | NA | <http://www.sph.umich.edu/csg/qin/HPeak/> | Perl |
| ChIP-Peak  analysis server | NA | <http://ccg.vital-it.ch/chipseq/> | Web interface |
| Bioconductor ChIPseq | NA | <http://www.bioconductor.org/workshops/2008/SeattleNov08/> | R |
|  |  |  |  |
| **Epigenetic Modifications Programs** | | |  |
| ChipDiff | 33 | <http://cmb.gis.a-star.edu.sg/ChIPSeq/paperChIPDiff.htm> | C |
| Chromasig | 34 | <http://bioinformatics-renlab.ucsd.edu/rentrac/wiki/ChromaSig> | Perl,C/C++ |
| SICER | 35 | <http://home.gwu.edu/~wpeng/Software.htm> | Python |
| Models 1-3 | 40 | <http://gbic.biol.rug.nl/supplementary/2009/ChromatinProfiles/> | R |
| CCAT | 41 | <http://cmb.gis.a-star.edu.sg/ChIPSeq/tools.htm> | C |
|  |  |  |  |
| **RNA Polymerase Programs** | | |  |
| Finite Mixture  Model | 31 | Not publicly released |  |
| Poisson Mixture Model | 32 | Not publicly released |  |

**Supplementary Reference:**

S1. Albert, I.*, et al.* GeneTrack-a genomic data processing and visualization framework. *Bioinformatics* **24**, 1305-6 (2008).
