# Supplementary material for: Evaluation of Algorithm Performance in ChIP-Seq Peak Detection
Source: PLoS One. 2010 Jul 8;5(7):e11471. doi: 10.1371/journal.pone.0011471 (PMC2900203; doi:10.1371/journal.pone.0011471)
Supplement: Table S2 — Methods used to rank peak lists from different programs. If programs returned a sorted peak list by default, no further sorting was conducted (NA). Secondary sorting method was used to break ties following the primary sorting. (0.03 MB DOC) [file pone.0011471.s002.doc]

**Supplementary Table 2.** Methods used to rank peak lists from different programs. If programs returned a sorted peak list by default, no further sorting was conducted (NA). Secondary sorting method was used to break ties following the primary sorting.

| **Program** | **Primary sorting** | | **Secondary sorting** |
| --- | --- | --- | --- |
| **PeakSeq** | NA (pre-sorted) | | |
| **CisGenome** | NA (pre-sorted) | | |
| **HPeak** | Maximum peak coverage |  | |
| **Sole Search** | # reads in peak | Effect size | |
| **MCPF** | # of reads in cluster |  | |
| **E-RANGE** | Fold enrichment | p-value | |
| **SISSRS** | Fold enrichment | p-value | |
| **MACS** | 10*-log_10_(p-value) | Fold enrichment | |
| **spp package**  **(wtd,mtc)** | False discovery rate | Score | |
| **QuEST** | q-value rank |  | |
