# Supplementary material for: Evaluation of Algorithm Performance in ChIP-Seq Peak Detection
Source: PLoS One. 2010 Jul 8;5(7):e11471. doi: 10.1371/journal.pone.0011471 (PMC2900203; doi:10.1371/journal.pone.0011471)
Supplement: Table S3 — Median and standard deviation of positional accuracy data. Median and standard deviation of the distance from estimated binding sites to the nearest high confidence motif occurrence, measured in base pairs. Measurements conducted for the top 1500 peaks in each peak list. Represented graphically in Figure 7 of the main text. (0.04 MB DOC) [file pone.0011471.s003.doc]

**Table S3.**  Median and standard deviation of the distance from estimated binding sites to the nearest high confidence motif occurrence, measured in base pairs. Measurements conducted for the top 1500 peaks in each peak list. Represented graphically in Figure 7 of the main text.

|  | **NRSF** | | **GABP** | | **FoxA1** | |
| --- | --- | --- | --- | --- | --- | --- |
| **Program** | **Median** | **SD** | **Median** | **SD** | **Median** | **SD** |
| **PeakSeq** | 0 | 82 | -2 | 73 | -2 | 103 |
| **CisGenome** | -0.5 | 24 | 2 | 64 | 1 | 74 |
| **HPeak** | 34 | 60 | 26 | 66 | 20 | 86 |
| **Sole-Search** | 1 | 35 | -1 | 61 |  |  |
| **MCPF** | 1 | 32 | -1 | 67 | 3.5 | 71 |
| **ERANGE** | 2 | 32 | 0 | 67 | 2 | 74 |
| **SISSRS** | 2 | 33 | -3 | 60 | 0 | 68 |
| **MACS** | 0 | 16 | -2 | 63 | 2 | 72 |
| **wtd** | 1 | 15 | -1 | 57 | 0 | 68 |
| **mtc** | 0 | 17 | -1 | 56 | 0 | 66 |
| **QuEST** | 12 | 16 | 12 | 60 | 1 | 87 |
