# Supplementary figures and images for: Evaluation of Algorithm Performance in ChIP-Seq Peak Detection
Source: PLoS One. 2010 Jul 8;5(7):e11471. doi: 10.1371/journal.pone.0011471 (PMC2900203; doi:10.1371/journal.pone.0011471)

**CisGenome**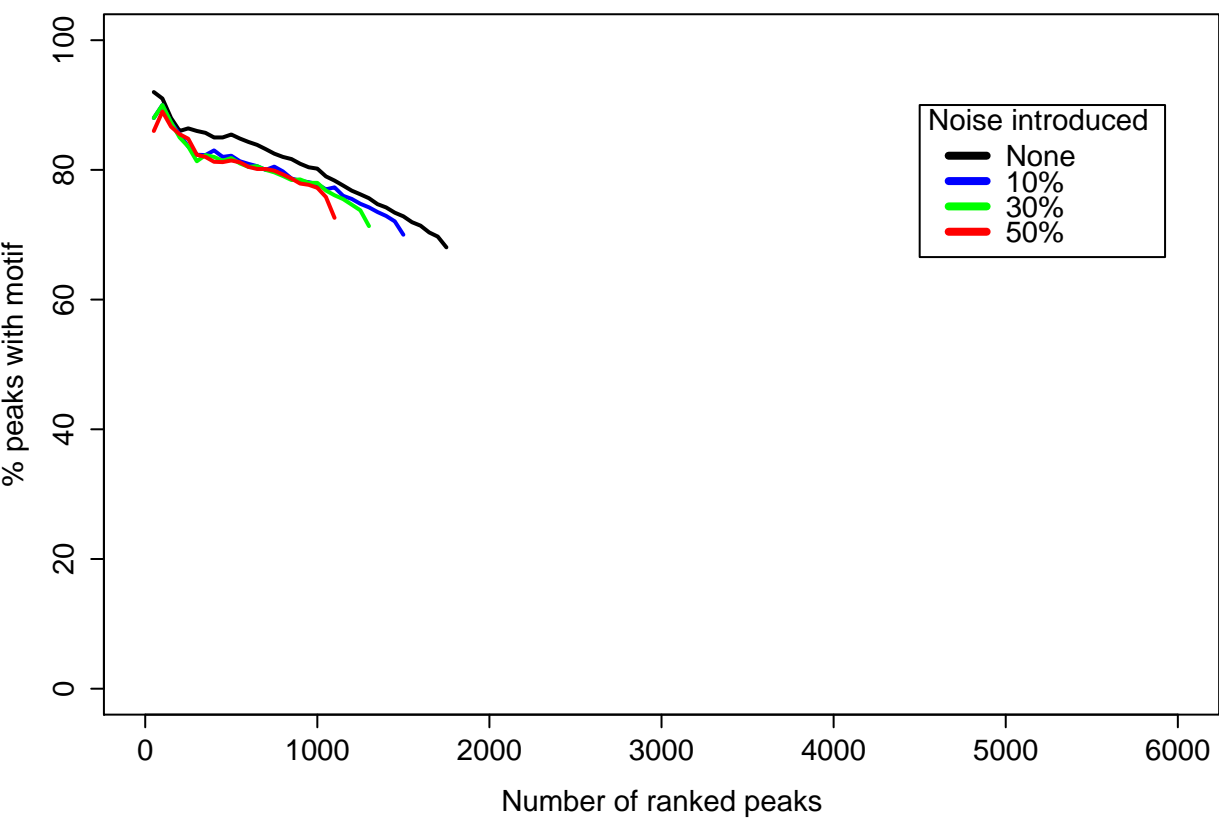**ERANGE**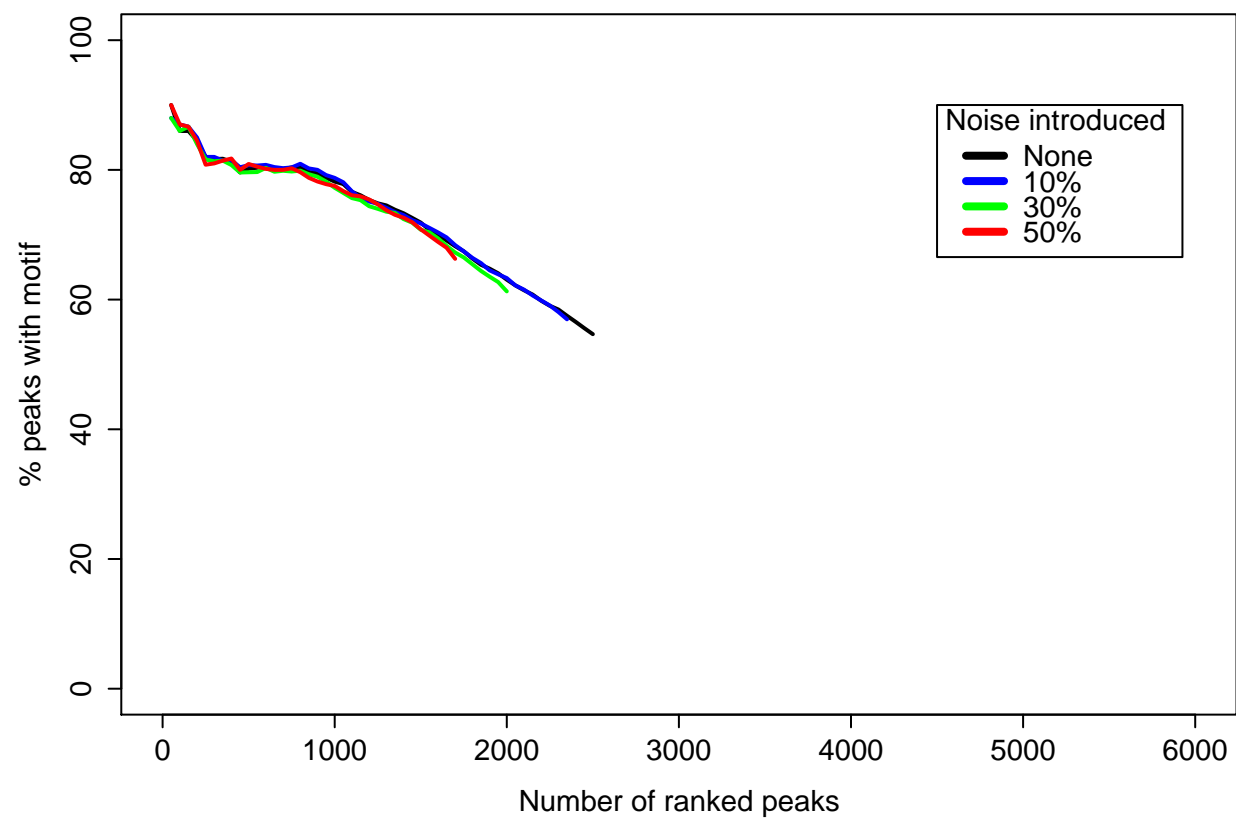**HPeak**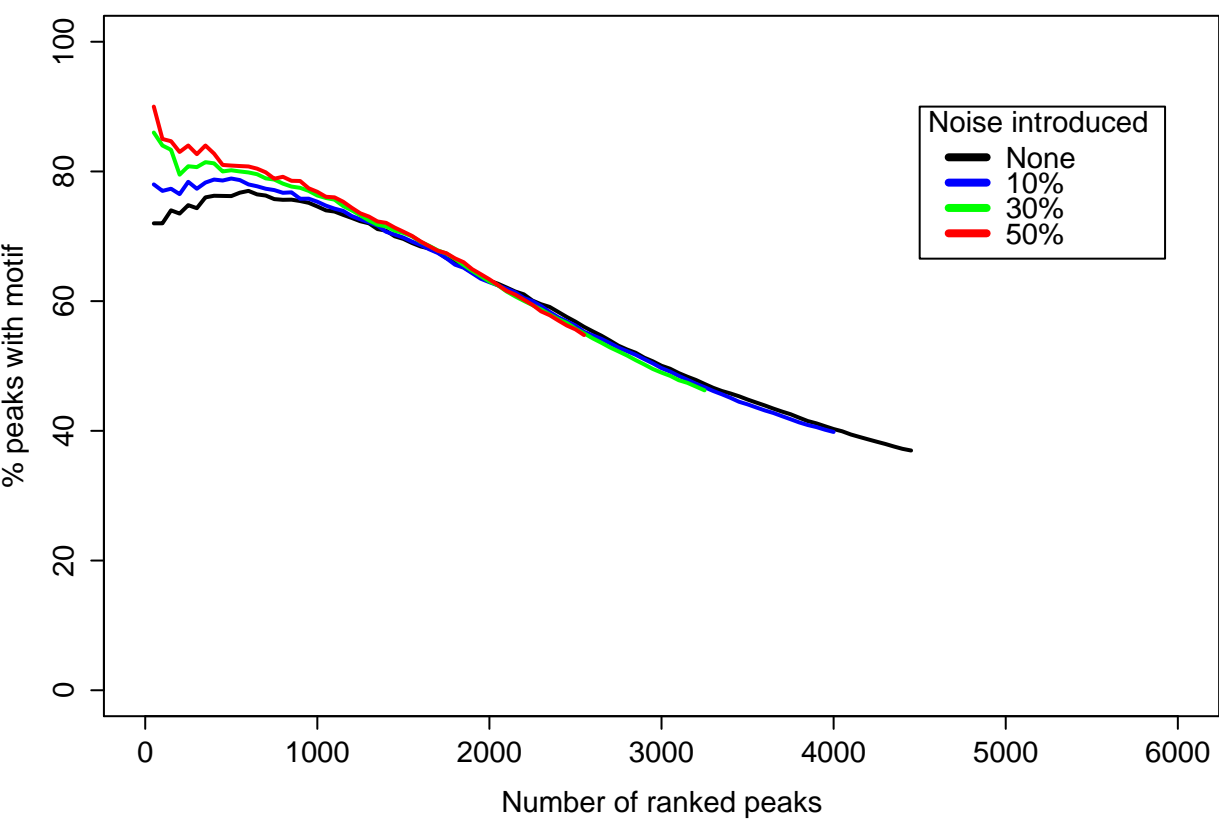**mtc**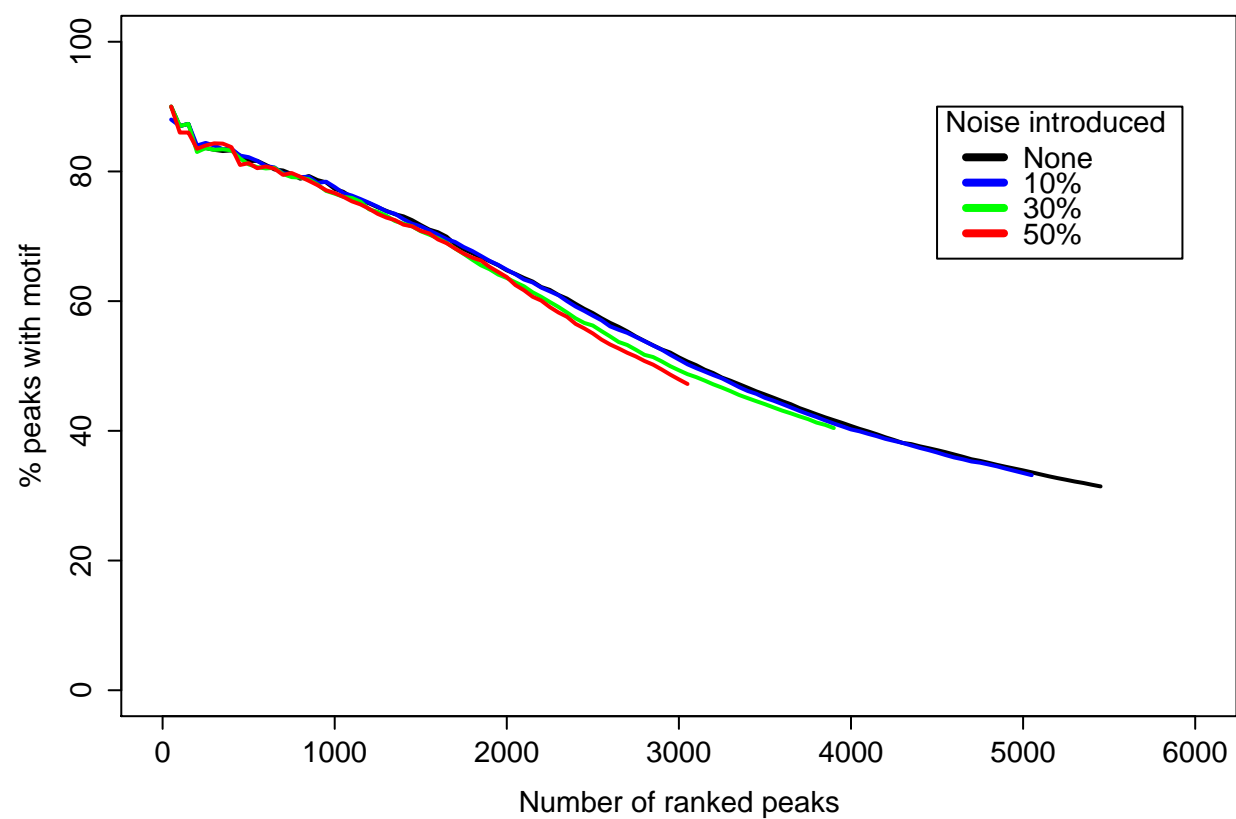**PeakSeq**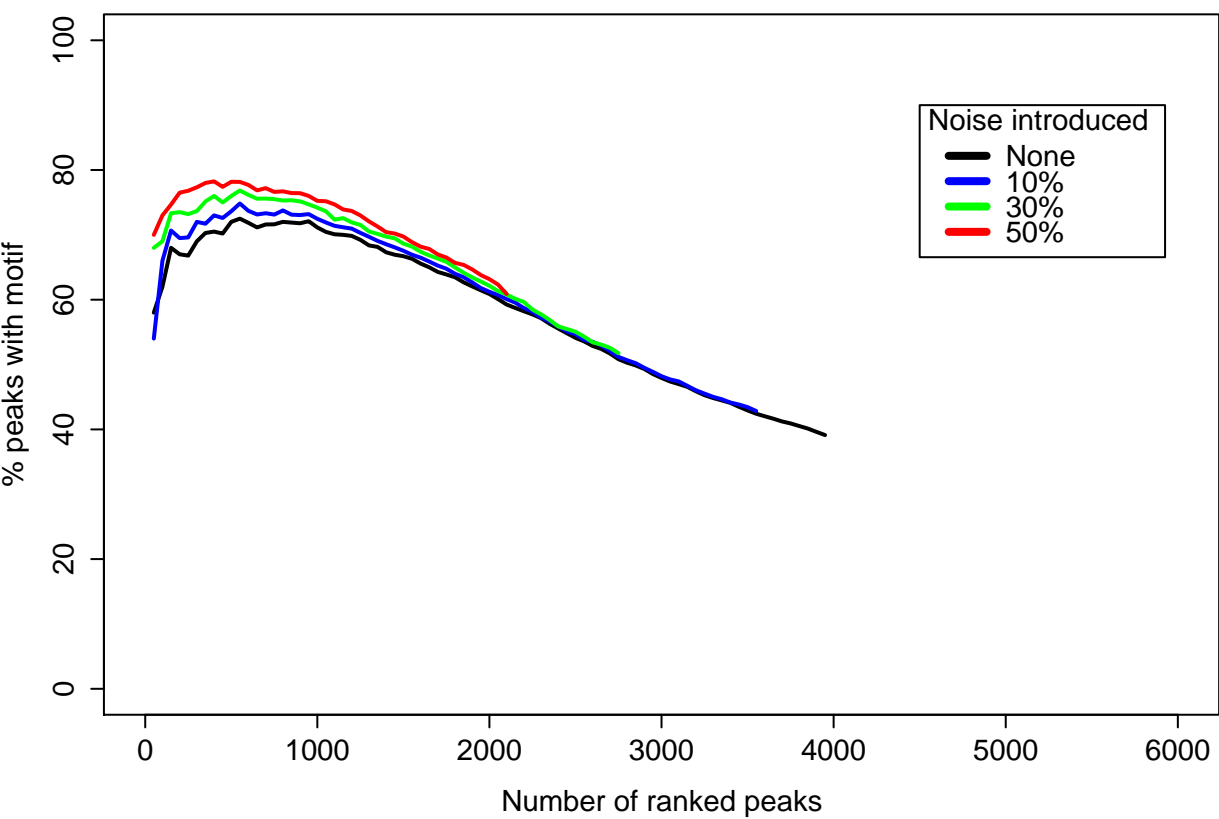**QuEST**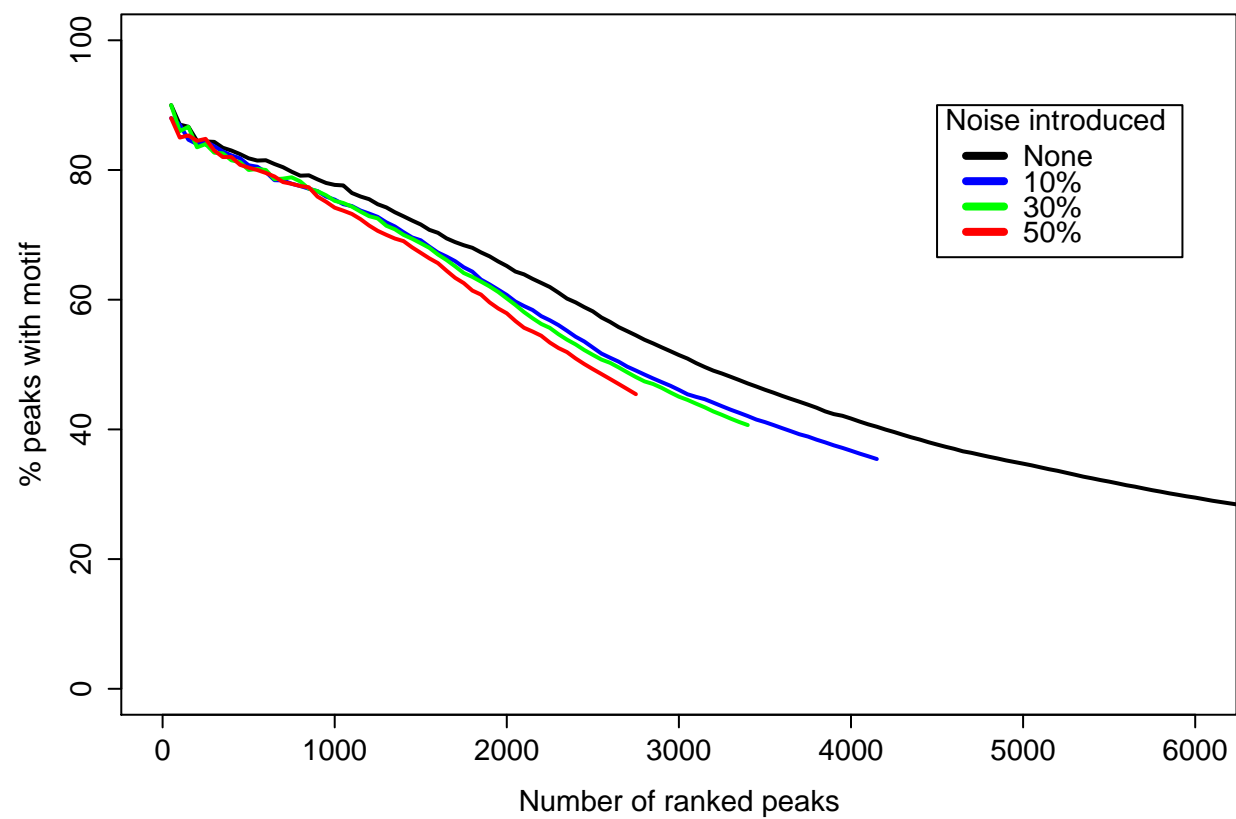

**SISSRS**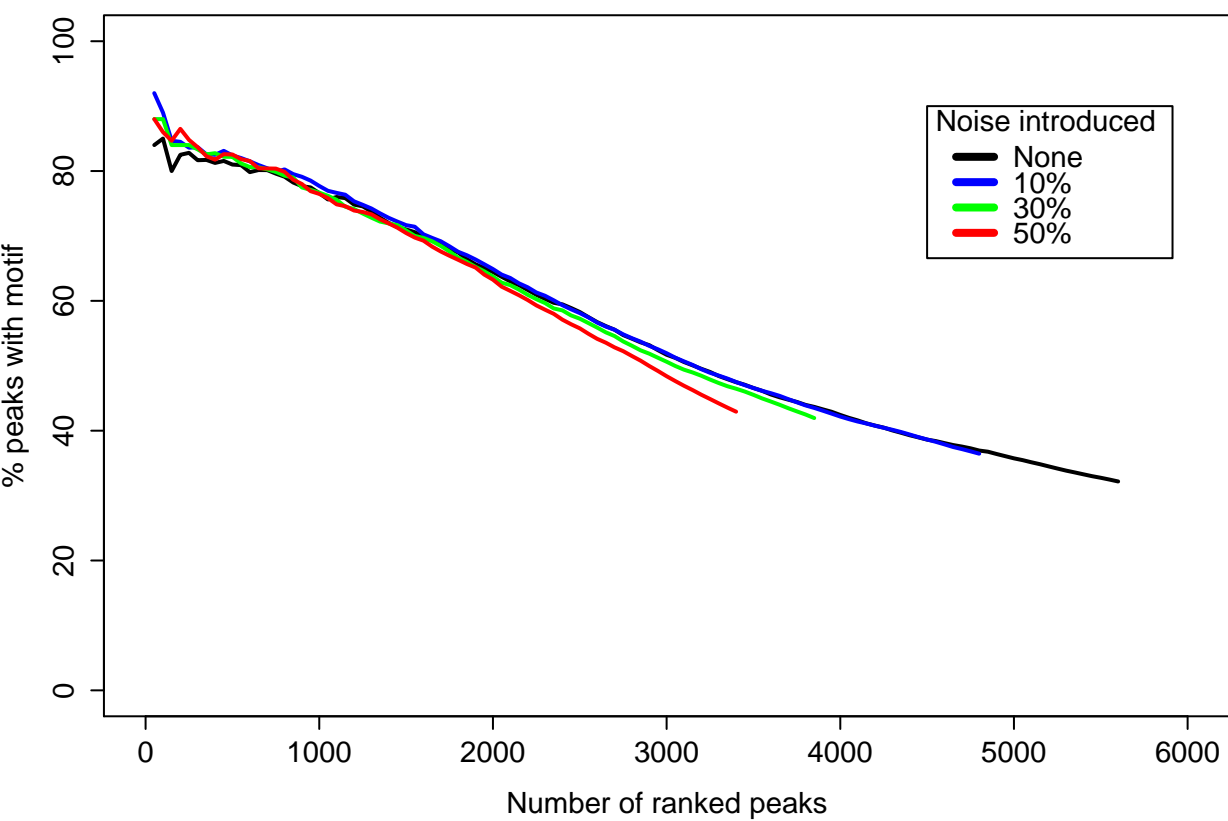**Sole-Search**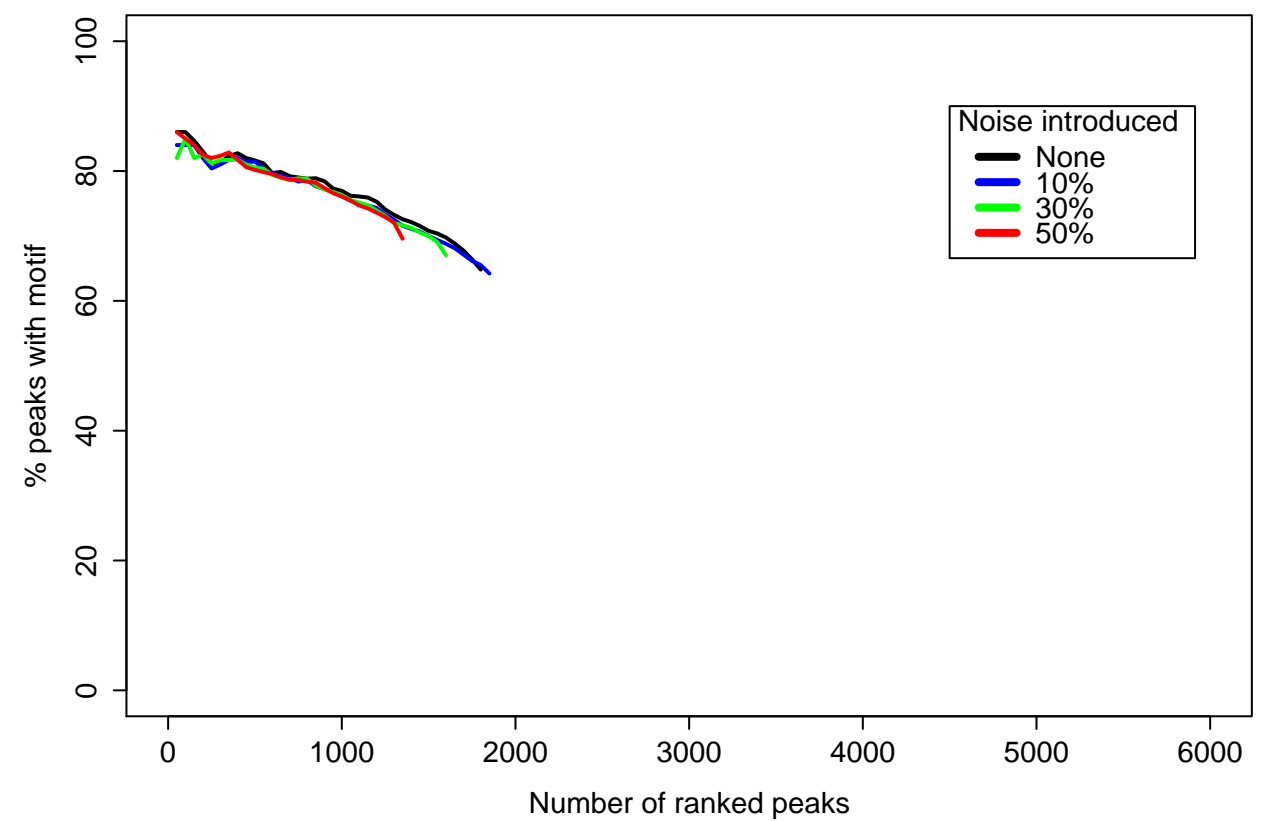**MCPF**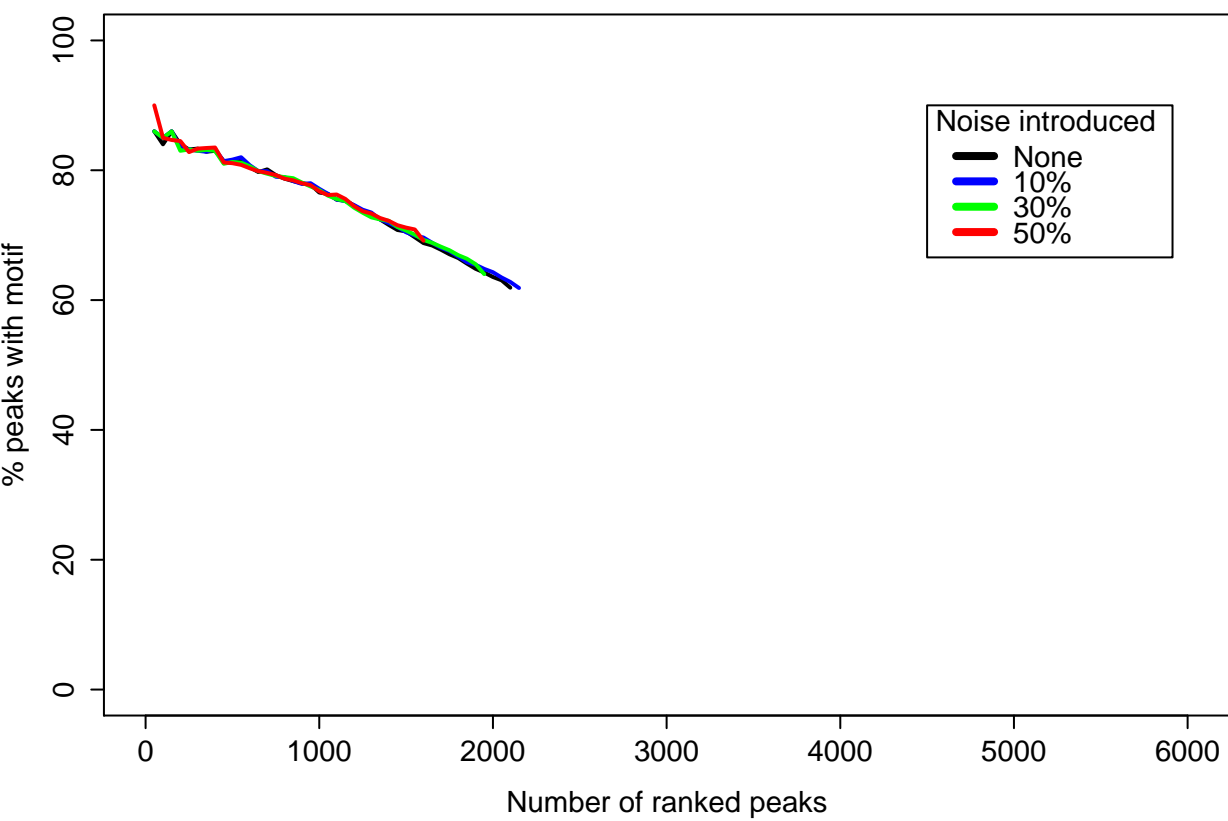**wtd**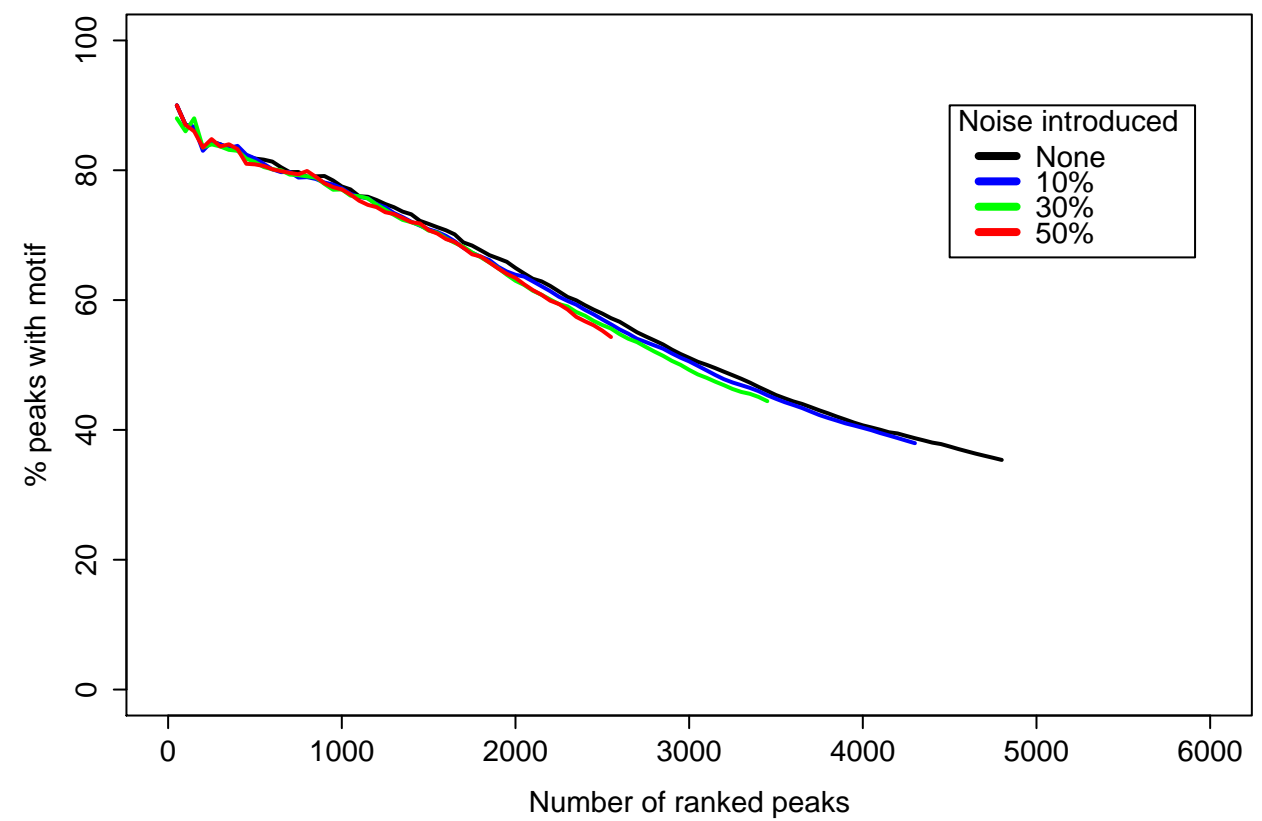**MACS**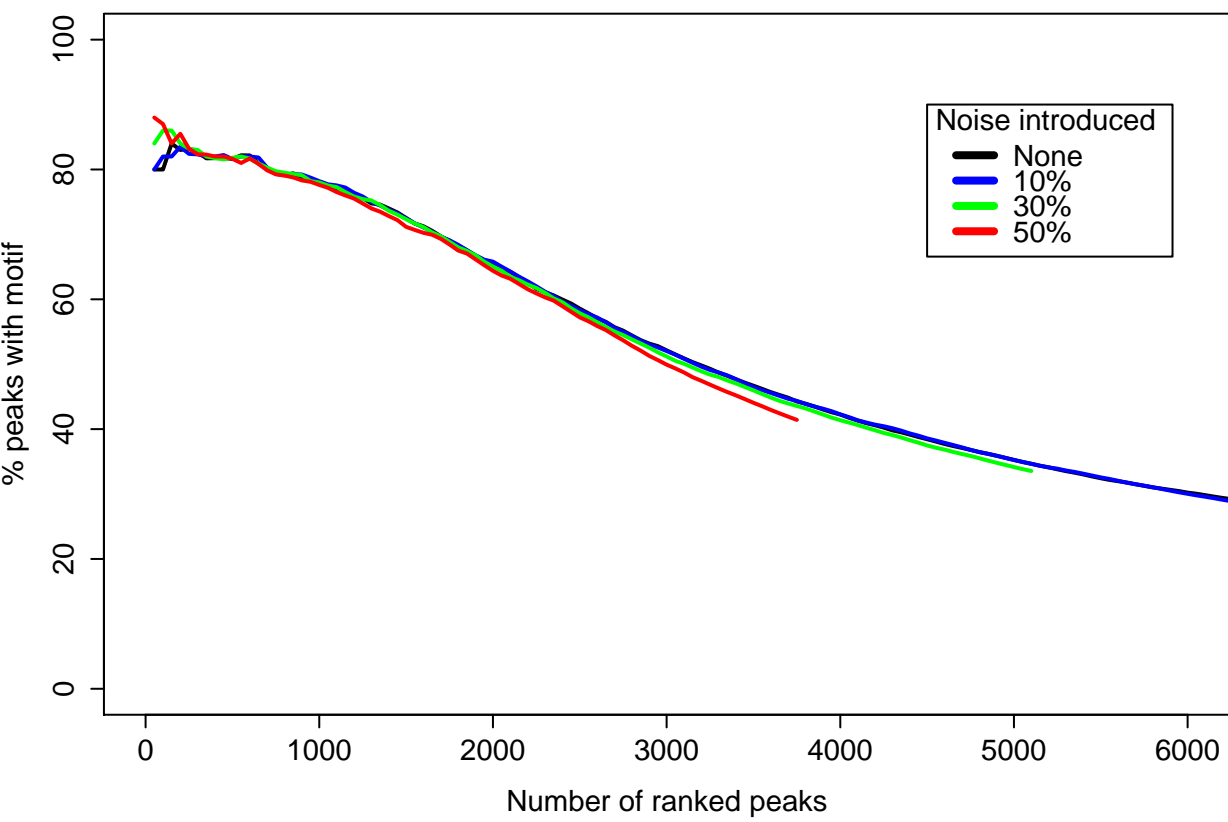

Supplement: Figure S7 — Motif content in ranked peaks from simulated noisy datasets. Panels show the change in motif content throughout the peak lists in Johnson et al. 's unpertubed ChIP sample and 10–50% noise introduction from background sequence for each program. (0.08 MB PDF) [file pone.0011471.s011.pdf]
